# Herbivory regulates the establishment of a native species of submerged aquatic vegetation (SAV) in a tidal estuary of the USA

**DOI:** 10.1007/s00442-019-04439-4

**Published:** 2019-06-22

**Authors:** A. J. Johnson, R. J. Orth, K. A. Moore

**Affiliations:** Department of Biological Sciences, Virginia Institute of Marine Science, William & Mary, PO Box 1346, Gloucester Point, VA 23062-1346 USA

**Keywords:** Plant population, Non-native, Restoration, Recovery, Blue crab

## Abstract

Herbivores are a diverse group of fauna that shape the distribution and composition of plant communities. In some cases, herbivory may prevent the re-establishment of submerged aquatic vegetation (SAV), such as *Vallisneria americana*, into systems. The goal of this study was to investigate the role and nature of herbivory on *V. americana* transplants with camera and transect surveys of grazing intensity and with field and laboratory grazing experiments using a suspected herbivore, the blue crab, *Callinectes sapidus*. Camera surveys recorded *C. sapidus* clipping and consuming shoots of *V. americana* for the first time. Grazing intensity surveys in low-salinity regions of the lower Chesapeake Bay indicated that the majority of *V. americana* transplants (50–75%) were clipped off at their base within one week of planting. Field and laboratory experiments demonstrated that *C. sapidus* clips and consumes *V. americana* as well as other rapidly colonizing, non-native SAV. Analysis of the gut contents of *C. sapidus* caught in SAV beds in the Chesapeake Bay revealed that SAV comprised 16% of their stomach contents, suggesting low levels of *C. sapidus* herbivory occurred over a wide area. *Callinectes sapidus* is yet another animal documented to consume SAV for some portion of their diet. These results also suggest that herbivores or omnivores, including *C. sapidus*, can serve as bottlenecks to recovery of SAV, like *V. americana*, in some areas. Herbivores may not serve as bottlenecks in other environments or to other SAV with more rapid plant growth or higher recruitment levels that may overcome grazing pressure.

**Electronic supplementary material:**

The online version of this article (10.1007/s00442-019-04439-4) contains supplementary material, which is available to authorized users.

## Introduction

Herbivores can influence plant community structure in both terrestrial and aquatic environments and subsequently the ecosystem services they provide (Cyr and Pace 1993; Burkepile 2013; Van Donk and Otte 1996; Green et al. 1997; Maron and Crone 2006). For a plant population to establish and persist in the presence of a robust herbivore community, it must develop mechanisms to withstand the grazing pressure in the system (Lodge 1991; Bakker et al. 2016; Scott et al. 2018). While the effects of herbivory on colonizing plant propagules may not initially be as evident as the effects of herbivory on established plant populations, the consumption of vulnerable, colonizing plant life history stages has long been hypothesized as especially important to the recruitment and dynamics of plant populations (Janzen 1970, 1971; Harper 1977). For example, in a meta-analysis of seedling mortality, herbivory was the most frequently recorded source of seedling mortality across plant species (Moles and Westoby 2004). For clonal submerged aquatic vegetation (SAV) populations, consumption of propagules that are important for both developing new populations and maintaining existing populations may be an important bottleneck to population growth or recovery (Rybicki et al. 2001; Eriksson and Ehrlen 2008; Orth et al. 2012).

Wild celery, or *Vallisneria americana* (Michx), is a submerged angiosperm found in tidal and non-tidal freshwater habitats throughout North America and is widely consumed across this range by turtles, waterfowl, and crayfish (Lodge and Lorman 1987; Lodge 1991; Sponberg and Lodge 2005). *Vallisneria americana* is a meadow-forming species that grows long ribbon-like leaves from shoots near the sediment surface. As a dioecious, clonal plant species, *V. americana* individuals are capable of both sexual and asexual reproduction (Sculthorpe 1967). Female flowers of *V. americana* are fertilized at the water surface and eventually produce fruits, each capable of dispersing 100–300 seeds (Lokker et al. 1997; Jarvis and Moore 2008). Individual shoots of *V. americana* reproduce asexually through stolon production and in northern habitats produce over-wintering buds. Both asexual reproduction and sexual reproduction are, thus, potentially important in the persistence, expansion, and recovery of *V. americana* populations.

Within the tidal freshwater and oligohaline regions of the Chesapeake Bay estuary, watershed inputs of nutrients and sediments in the twentieth century lowered water quality and substantially reduced SAV populations (Moore et al. 2000; Cercro and Moore 2001; Kemp et al. 2005). In one region, encompassing the upper areas of the tidal James and Chickahominy Rivers, these nutrient and sediment loadings resulted in dramatic declines in native SAV, including *V. americana* (Moore et al. 2000). Areas historically vegetated with *V. americana* and other native SAV remain either unvegetated or are now colonized with mixtures of non-native vegetation such as *Hydrilla verticillata* (L.f. Royle, “hydrilla”) or *Najas minor* (All., “spiny naiad”) (Orth et al. 2017). Because *V. americana* has a wide salinity tolerance, 0–15 (Doering et al. 2001; Martin and Valentine 2012), and was historically abundant in the estuary throughout this salinity range, it has been the focal species for SAV restoration within the tidal freshwater and oligohaline environments of the James and Chickahominy Rivers. These experimental restoration attempts using both single adult shoots and seedlings in transplant garden plots have, to date, been largely unsuccessful. Restoration failure has been attributed to aquatic herbivory of unprotected propagules (Moore et al. 2010). In contrast, adult plants and seedlings of *V. americana* survived and grew within enclosures protecting *V. americana* from potential herbivores (Meier 2002; Moore et al. 2010). These results point to herbivory as the critical bottleneck to *V. americana* recruitment and recovery within the tidal James and Chickahominy Rivers.

The goal of this study was to better understand the specific nature and role of herbivory limiting the re-establishment and restoration of this native, freshwater plant species into its original habitat. Specific objectives were: 1. To identify the primary herbivores consuming *V. americana* shoot propagules within the system; 2. To determine the grazing intensity of the herbivore community on individually planted *V. americana* propagules; 3. To evaluate the grazing intensity of a suspected generalist omnivore, the blue crab, *Callinectes sapidus*, on *V. americana* relative to a non-native SAV species present in the system; and finally, 4. To evaluate the gut contents of *C. sapidus* individuals collected from the James and Chickahominy Rivers to determine if *C. sapidus* outside experimental trials consumed vegetation.

## Methods

### Study design

The study was conducted over two consecutive years, 2016 and 2017. In late summer (August–October) 2016, a field survey using underwater photography was conducted to identify potential *V. americana* herbivores adjacent to restoration plots in the James and Chickahominy Rivers, in the lower Chesapeake Bay, VA. In addition, *V. americana* vegetative propagules (transplants) were planted along transects over three trials to evaluate grazing intensity after 1 and 7 days at these same locations. After analyzing and interpreting the results from these surveys, in situ caging experiments were conducted in 2017 to specifically evaluate the grazing effects of *C. sapidus*, on transplants of *V. americana.* Because *C. sapidus* was the only herbivore observed both during these surveys and in another previous study of SAV herbivory conducted in this region (Meier 2002), it was chosen for more detailed study. Additional laboratory experiments were then conducted to compare consumption by *C. sapidus* between *V. americana* and a non-native species, *H. verticillata*, which is present and abundant in this region and other tidal, freshwater and oligohaline portions of Chesapeake Bay. Lastly, wild *C. sapidus* was collected near the experimental sites in the lower Chesapeake Bay to identify their gut contents outside an experimental setting. Nursery grown vegetative transplants were used in all experiments. Prior research (Moore et al. 2010) at the sites noted here showed vegetative transplants and seedlings were consumed equally allowing us to use vegetative transplants as proxies for seedlings. Before transplanting, all *V. americana* and *H. verticillata* individuals were scraped clean of any obvious epiphytes. All applicable institutional and national guidelines for the care and use of animals were followed.

### Study sites

**Fig. 1:**
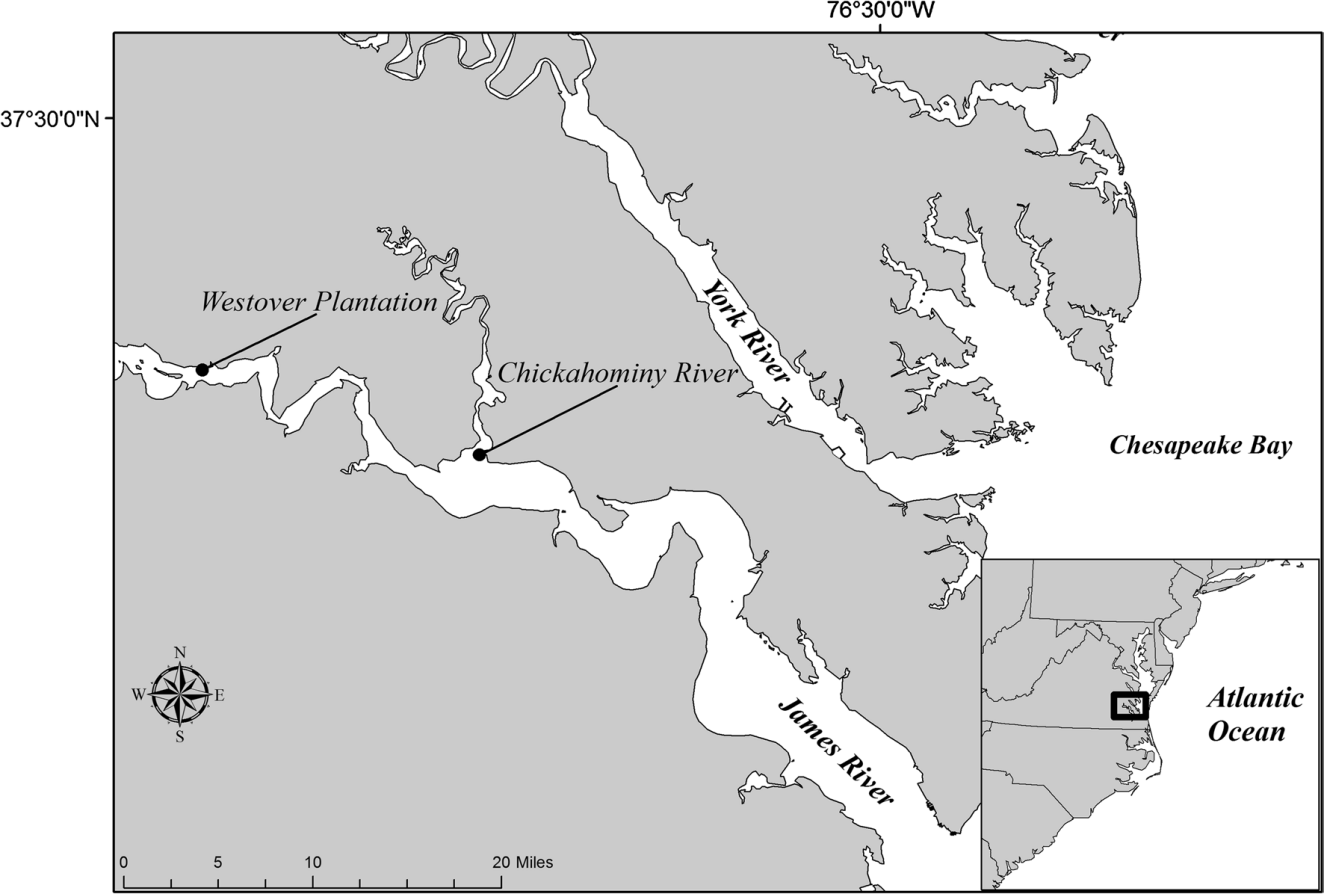
The location of experiments and surveys throughout the tidal, freshwater James River and Chickahominy River, Virginia

Locations in the James (37.310699, − 77.155512) and Chickahominy Rivers (37.263984, − 76.873465), VA were chosen because they historically supported stable SAV populations and are both locations of largely unsuccessful *V. americana* restoration efforts (Fig. 1). Sites within the James River currently have no persistent SAV, while sites within the Chickahominy River have fringing and seasonally persistent meadows of two non-native SAV species, *N. minor* and *H. verticillata*. Field surveys, transplant herbivory surveys, and in situ caging experiments were conducted at depths  ≤ 0.5 m MSL at these sites.

### Herbivore identification

A field survey using underwater photography was conducted in the James and Chickahominy Rivers, in late summer 2016 to identify herbivores most likely consuming *V. americana* transplants and seedlings. Four GoPro^®^ cameras set to photograph at one-second intervals were deployed 8 cm from 3 to 4 transplanted *V. americana* shoots. Cameras were deployed eleven times in August and September 2016 for ~ 2 h. Due to camera malfunctions, obstructions to the field of view, and poor visibility, the duration of usable photography from a camera deployment varied among sampling events. This survey was conducted on three separate deployments in the James River for a total of 24 h of footage. Within the Chickahominy River, the survey was conducted on eight separate deployments for a total of 54 h of footage. More cameras were deployed in the Chickahominy River after determining photographs in this area were consistently and reliably of higher quality than at the James River location, and the observed clipping of shoots ~ 2 cm above the meristem suggested that the same herbivore was present at both locations. All recordings were conducted on rising tides (~ half an hour after low) in case the herbivore was more active in deeper water. Photographs were inspected for any interactions, or physical engagement, with *V. americana* shoots. The total number of animals in the field of view and the number of animals directly interacting with (identified as touching, damaging, clipping, or biting) the transplants in the photographs were counted and identified to determine the most likely *V. americana* consumers.

### Grazing intensity

**Fig. 2:**
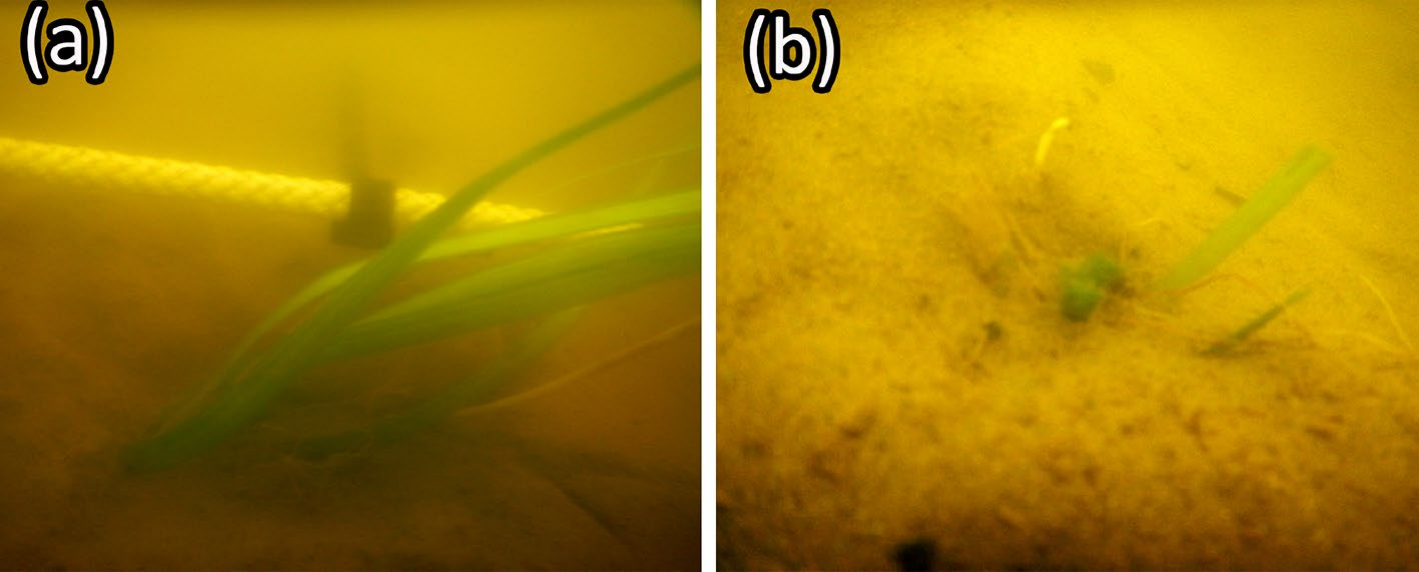
**a** A *V. americana* transplant planted every 0.5 m along the 10-m transect used for grazing intensity surveys in the James and Chickahominy Rivers. **b** A clipped *V. americana* transplant along a transect at the mouth of the Chickahominy River

To quantify the intensity of *V. americana* consumption within the James and Chickahominy Rivers, one shoot of *V. americana* with at least 10 cm leaves was transplanted every half meter along a 10 m unvegetated transect at each location. All transplants were then inspected for herbivory after 1 and 7 days. In total, 20 shoots were transplanted at each site along the transect for a given trial. A 10-m guide rope was laid between two PVC stakes with marks every 0.5 m to indicate a transplant location. Transplants were planted ~ 2–3 cm within the sediment. After planting, the composition and percent cover of SAV within a meter of the planting line were determined visually every meter. Transplants were considered grazed if they were clipped to ~ 2–4 cm height, the characteristic mark of the dominant grazer within these systems (Fig. 2). Missing shoots were labeled as such to distinguish between transplants whose leaves had been clipped (“grazed”) and those who may have been consumed or lost by other means (“missing”). This procedure was repeated for three separate trials at each location in 2016. An additional transect trial was placed within a densely vegetated *N. minor* meadow (~ 95% bottom cover) in the Chickahominy River in 2016 to gauge if herbivory occurred within existing SAV in the system. Three additional transect trials were conducted at the same location in the Chickahominy River in summer 2017 to test if grazing intensity varied at this location between 2016 and 2017.

### *Callinectes sapidus* grazing intensity

To directly estimate the grazing intensity of a potentially important herbivore, *C. sapidus*, on *V. americana*, five, circular 0.06 m^2^ aluminum wire (2 mm diagonal mesh size) cages were used to contain individual *C. sapidus* with two *V. americana* transplants for 72 h in situ within the Chickahominy River (Fig. S1). An aluminum wire cage not containing *C. sapidus* and an uncaged control, each also containing two *V. americana* transplants, were constructed adjacent to each caged *C. sapidus* treatment to form a block containing one experimental unit of each treatment. Each transplant was cut to 20-cm leaf length, and the number of intact leaves was counted. The location of each transplant within the cage relative to shore was also recorded to track consumption of each transplant in each cage. Individuals of *C. sapidus* ranged in carapace width (CW) from 2.5 to 17.5 cm. Cages were constructed with aluminum wire (height = 40 cm) attached to plastic cylinder (height = 15 cm) with a 48” cable tie. At deployment, the plastic cylinder was pushed 8 cm into the sediment to prevent *C. sapidus* from burrowing out or into treatments and anchored in place with one, 2-cm PVC and one rebar stake. After 72 h, the length of all transplant leaves was measured and each leaf was inspected for bite marks. For each of the eight trials, five blocks were created, and each block contained all three treatments. These in situ cages excluded other potential herbivores from *V. americana* transplants but provided alternative food items, such as epifauna in the water column and infauna within the sediment, for *C. sapidus* within the cages. As a result, at the end of a given trial, cages were also visually inspected for any obvious alternative prey inhabiting them. Blocks of cages were placed at least two meters apart in bare sediment and in between clumps of the non-native, freshwater plants *N. minor* and *H. verticillata*, which are prevalent in the system. Five, 0.07 m^2^ sediment cores were taken and five, 2.5 m^2^ dip net pushes (2 mm diameter mesh) were made within a *N. minor* meadow adjacent to the experiments to estimate sediment infauna and epifauna in the *N. minor* meadow surrounding the cage experiment. In addition, five, 20 cm W × 80 cm L mesh (500 μm) epifaunal bag samples (similar to Duffy et al. 2015) were taken from *N. minor* patches in between the blocks of cages to further categorize the epifaunal community in the area. All epifaunal bag samples were emptied into plastic bags and frozen until contents could be identified in the lab.

### Non-native SAV consumption

To gauge if *C. sapidus* consumes non-native SAV present in the system at a similar rate to the native plant *V. americana*, *C. sapidus* was collected from the Chickahominy River on eight occasions in fall 2016 and placed in tanks with transplants of either *V. americana* or *H. verticillata* for 72 h. Eight 100-L tanks filled to 25 cm were placed into an 1800-L tank filled with recirculating water chilled to 24 °C. Four vegetative transplants of *V. americana* or *H. verticillata* were planted in each 100-L tank. The number of *V. americana* leaves or *H. verticillata* shoots was counted for each transplant and the length of all transplants was cut to 20 cm before planting. A single *C. sapidus* was introduced into two of the tanks planted with *V. americana*, and a single *C. sapidus* was introduced into two of the tanks planted with *H. verticillata*. The remaining four 100-L tanks, two tanks per plant species and each containing four transplants of the respective species, received no *C. sapidus* and served as crab-less controls. Twenty-four hours after introducing *C. sapidus*, the plant transplants were inspected and any uprooted transplants were replanted as any uprooting over this time period may potentially have resulted from *C. sapidus* acclimation to the tank environment. Seventy-two hours after introducing *C. sapidus* into the tank, the length of each remaining leaf/shoot on a transplant was measured. Leaves of *V. americana* were also inspected for signs of tearing or biting. Suspected marks were categorized as “minimal” (> 1 mm but < 10 mm) or “heavy” (> 10 mm). Four trials were conducted with “large” *C. sapidus* (CW > 8 cm) collected with un-baited crab pots, and four trials were conducted with smaller *C. sapidus* (CW < 8 cm) collected with a 50-cm mouth dip net (2 mm diameter mesh). Collected *C. sapidus* ranged in size from 2 to 16 cm CW. In addition, at the end of each experiment, *C. sapidus* larger than 3 cm were removed from tanks (*n* = 24) and frozen for gut analysis to verify consumption of plant material had occurred.

### *Callinectes sapidus* diet survey

Gut contents were identified for *C. sapidus* collected by seining at two locations on either side of the experimental area at the mouth of the Chickahominy River, as well as across from restoration plots at Westover Plantation in the James River. Sampling occurred from July to September, 2017, on five occasions during the peak biomass of SAV in the region (Moore et al. 2010). Two replicate seines (30 m L x 1.2 m H, with 0.64-cm mesh) were made at each site during each sampling round. Each replicate seine was pulled over the same area but was separated by a minimum of 30 min. For each seine pull, the net was pulled out perpendicular to shore until fully extended or a depth of 1.2 meters was reached, at which point the offshore end of the seine was pulled down-current back to shore.

All captured *C. sapidus* were placed immediately on ice to reduce digestion of stomach contents until frozen. In the lab, the carapace width, sex, and any apparent damage to the crab were recorded before foreguts were dissected. The percent fullness of foreguts was then estimated as the displacement volume of a foregut when placed in either a 10 or 25 mL graduated cylinder filled with water, depending on the size of the foregut (see Seitz et al. 2011 for further discussion of methods). Each foregut was then emptied into a petri dish containing water and allowed to settle for 1 h at which point the relative contribution of amphipods, clams, copepods, crabs, gastropods, isopods, ostracods, polychaetes, shrimp, and plant matter to stomach fullness were estimated.

## Statistical analyses

### Grazing intensity

A generalized linear model (GLM) fit to a quasi-binomial distribution was constructed to determine if the location or time period after planting during a grazing intensity trial influenced the number of grazed transplants observed along transects in 2016. A separate GLM, also fit to a quasi-binomial distribution, was then used to compare the grazing intensity along transects at the mouth of the Chickahominy River between 2016 and 2017. The specific transect trial during which survival was evaluated was included as an additive term in each model to account for any temporal variability associated with grazing intensity at each location over the course of the three survey trial periods. Models were fit to quasi-binomial distributions to account for any potential overdispersion within the observed data. Model fit was evaluated graphically.

### *Callinectes sapidus* grazing intensity

A linear mixed-effects model was constructed to determine if the change in total leaf length for transplants in cages containing *C. sapidus* was significantly different to the change in total leaf length for transplants in control cages without *C. sapidus* or uncaged transplants exposed to the entire herbivore community after 72 h. Physical damage to cages resulting from boat wake and the availability of *C. sapidus*, caught within unbaited crab pots within the Chickahominy River but outside the experimental area, resulted in uneven blocks of treatments between trials. As a result, data were used for only thirty-one blocks containing all three treatments over the eight trials instead of the forty originally constructed blocks. The trial during which a given set of treatments was evaluated and the block within which a cage was situated was considered as nested, random terms in this model to account for any random spatial or temporal differences in grazing at the sampling location. The difference in total leaf length response variable was square-root transformed to meet model assumptions. Post hoc Dunnett’s multiple comparisons of least square means were conducted to evaluate differences in change in total transplant leaf length specifically between transplants inside cages containing *C. sapidus* and transplants planted outside cages. A generalized linear model was then used to establish if the estimated percentage of plant matter in a *C. sapidus* stomach was related to the difference in transplant leaf length within a given cage.

### Non-native SAV consumption

A linear mixed-effects model was used to compare the change in total length of *V. americana* or *H. verticillata* transplants in experimental tank systems with or without *C. sapidus* after 72 h. The presence or absence of *C. sapidus* and the species of SAV present in the tank were treated as interactive terms in the model, while the size of the *C. sapidus* added to the tank during a trial was considered as a separate fixed factor. The individual trial during which a *C. sapidus* was introduced to tanks was treated as a random factor to account for any variability resulting from successive trials. Categorical classifications of bite marks were analyzed with odds ratios to determine if the odds of observing tear or bite marks on *V. americana* differed between tanks with and without *C. sapidus*. Fisher’s exact tests were then used to estimate if the observed frequencies of tear or bite marks were significantly different than expected frequencies of marks (i.e. no difference in tearing or biting between crab and control tanks). A generalized linear model was then used to establish if the estimated percentage of plant matter in a *C. sapidus* stomach from a given tank was related to the difference in leaf or shoot length within that tank.

A type I error rate of 0.05 was established for all statistical tests. Generalized linear models and linear mixed-effects models were built with the *glm* and the *lmer* function from the *lmerTEST* R package (Kuznetsova et al. 2014). Post hoc Dunnett’s multiple comparisons of least square means were conducted with the *contrast* function in the *lsmeans* package (Lenth 2015). All statistics were performed in R statistical analysis software (R Development Core Team 2019).

## Results

### Identifying herbivores

**Fig. 3:**
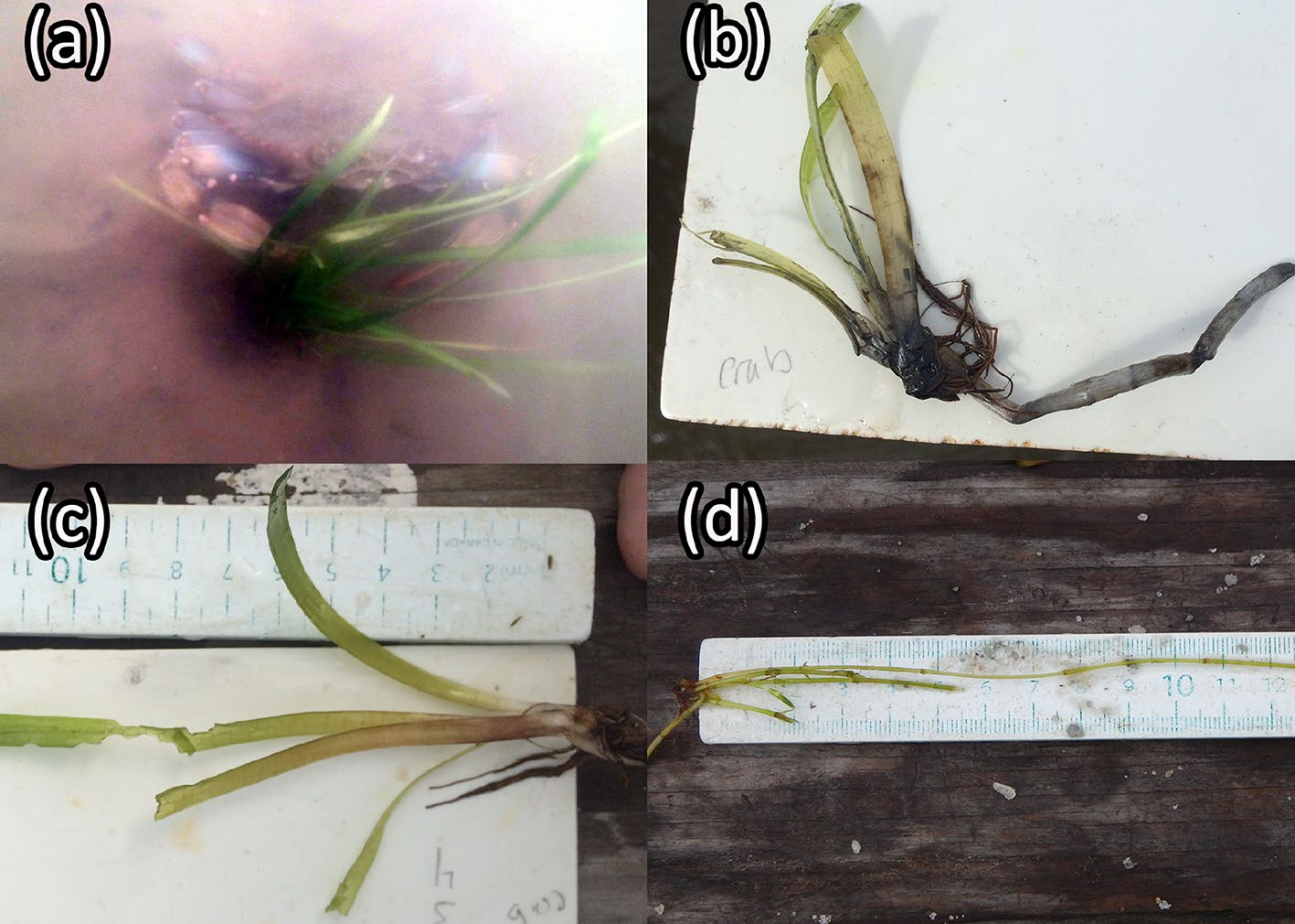
The signs of blue crab herbivory observed in situ in the James and Chickahominy Rivers and within laboratory experiments: **a**  a *C. sapidus* photographed interacting with a *V. americana* transplant in the Chickahominy River; **b** a clipped *V. americana* shoot from a cage containing one blue crab from the in situ caging study conducted in the Chickahominy River; **c** a *V. americana* shoot with a bite mark categorized as “heavy” (> 1 cm); and **d** a shoot of *Hydrilla verticillata* removed from a tank containing one blue crab. All of the whorled leaves, normally 5 per node, have been stripped from the shoot and several shoots have been clipped

Similar herbivore species assemblages were recorded in the Chickahominy River as in the James River. The most common species identified (Table S1) were tessellated darters (*Etheostoma olmstedi*), juvenile sunfish (*Lepomis* sp.), and blue crabs (*Callinectes sapidus*). Only *C. sapidus* was observed interacting with *V. americana* transplants (Fig. 3a). *Callinectes sapidus* interacted with transplants by grabbing leaves on six separate occasions, damaging transplants on two occasions by clipping leaves, and consuming a transplant leaf on one occasion (Video S1 shows the time-lapse photography of this consumption).

### Grazing intensity

**Fig. 4:**
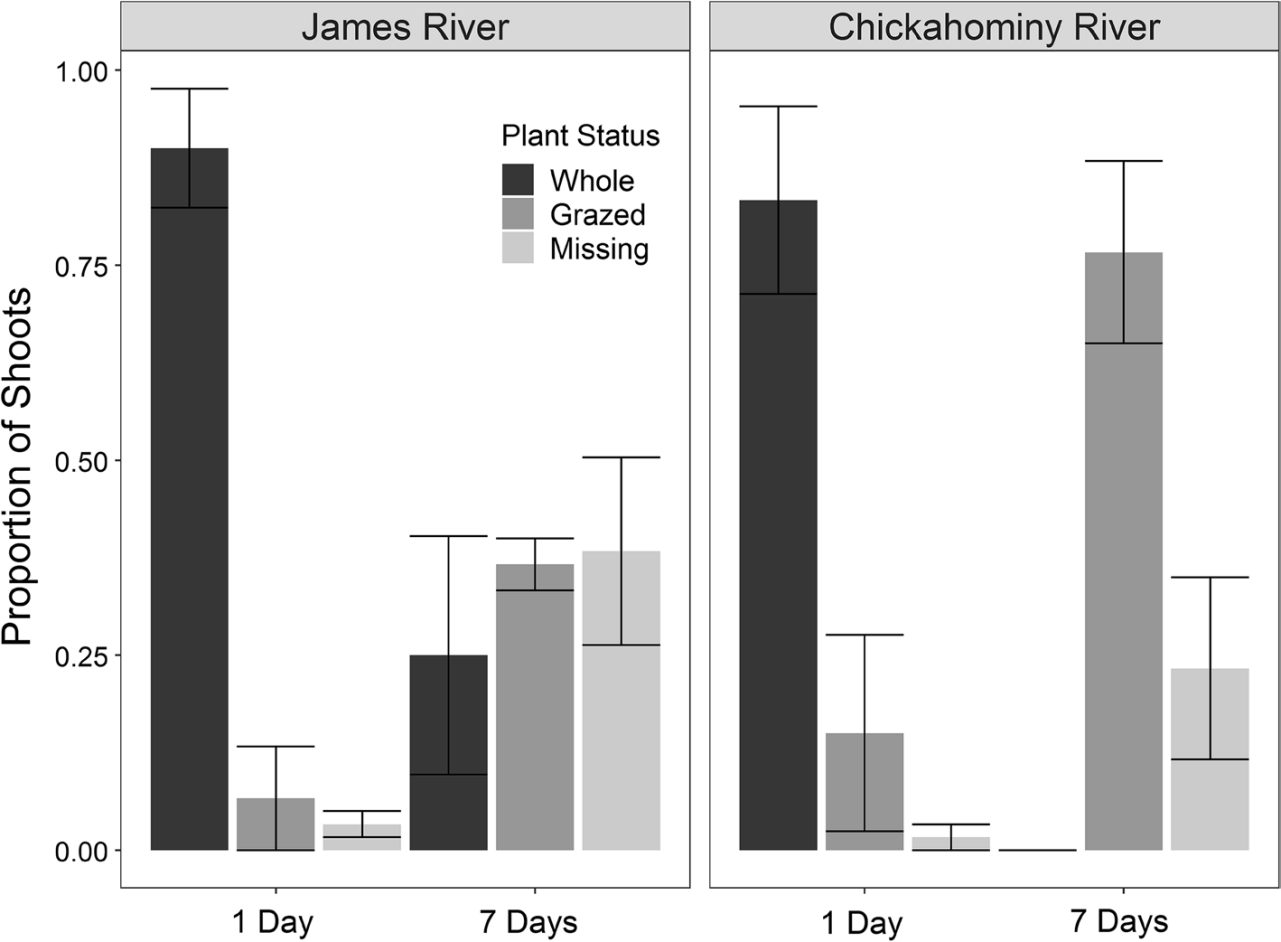
The mean proportion of shoots (± SE, *n* = 3 trials) whole, consumed (grazed), or missing after 1 day and 7 days along transects (20 shoots per transect) in the James and Chickahominy Rivers in late summer 2016

Significantly more transplants were consumed within seven days of planting than within one day of planting (*β* = 9.3 ± 1.7, *P *< 0.001, Fig. 4). On average, < 25% of the transplants were grazed after 1 day but 40–75% were grazed within seven days at both locations. No significant differences in transplant grazing were detected between locations (*p* = 0.1) and no significant interaction term was detected (*p* = 0.2). Grazing intensity was significantly different among the three successive trials (Table S2). Similarly, grazing intensity over the duration of a trial interacted significantly with the year of sampling in the Chickahominy River (*β* = 0.08 ± 1.9, *P *< 0.001, Fig. S2 and S3). Although diagnostics of this generalized linear model describing grazing intensity between 2016 and 2017 suggest a poor model fit, data visualization corroborates model results (Fig. S3) and generally suggests that grazing occurred in both 2016 and 2017, but that the duration over which a transplant experienced this grazing differed between the 2 years. Regardless of the year or location, however, no transplants survived until the end of the growing season. At the end of the six successive sampling weeks in 2017, for example, only 3 of the 60 total planted shoots remained ungrazed (5%) and none survived. The additional transect placed within a *N. minor* meadow in 2016 exhibited similar herbivory trends to adjacent transects placed in sediment with lower *N. minor* cover, with 75% of shoots intact after 24 h and only 30% remaining after 1 week (Fig. S4). Grazing recorded along transects in 2017 also compliments this finding, as *N. minor* was present along previously bare sediment transects at the mouth of the Chickahominy River in 2017.

### *Callinectes sapidus* grazing intensity

**Fig. 5:**
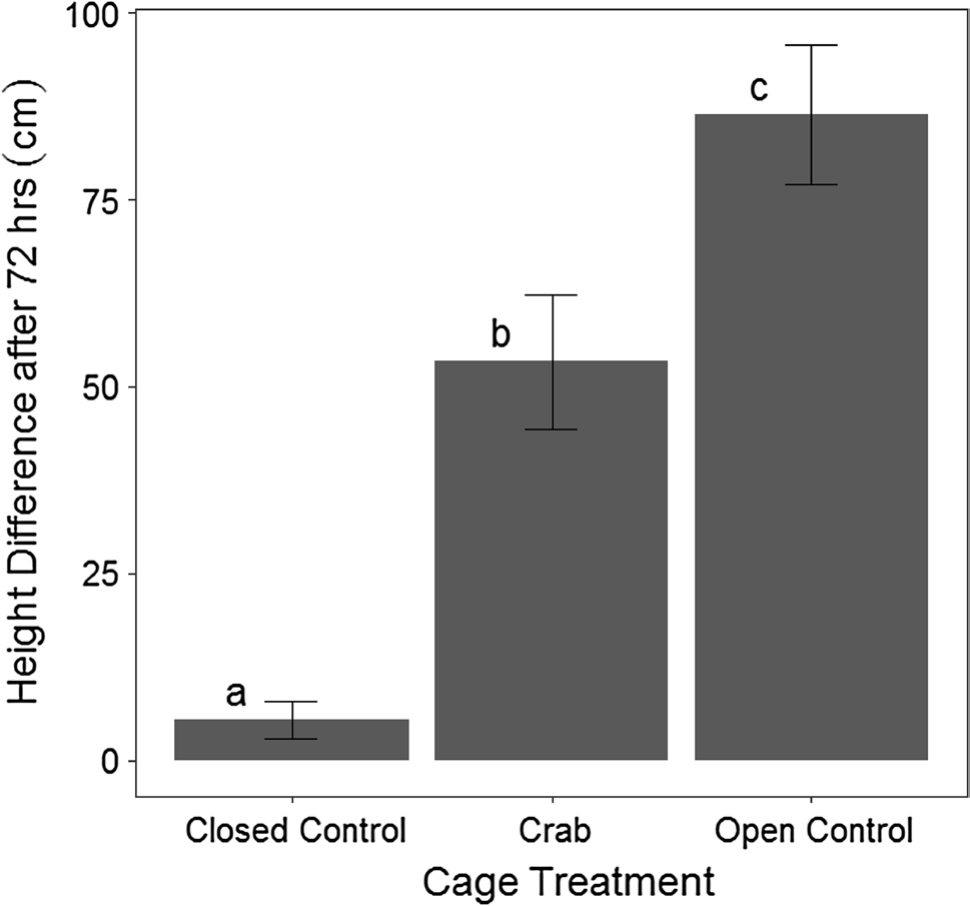
The mean difference in leaf length per cage (± SE) for *V. americana* shoots planted in cages (*n* = 31) without *C. sapidus* (closed control), with *C. sapidus* (Crab), and outside any cage (open control). Letters indicate significant differences (*p* < 0.05)

**Fig. 6:**
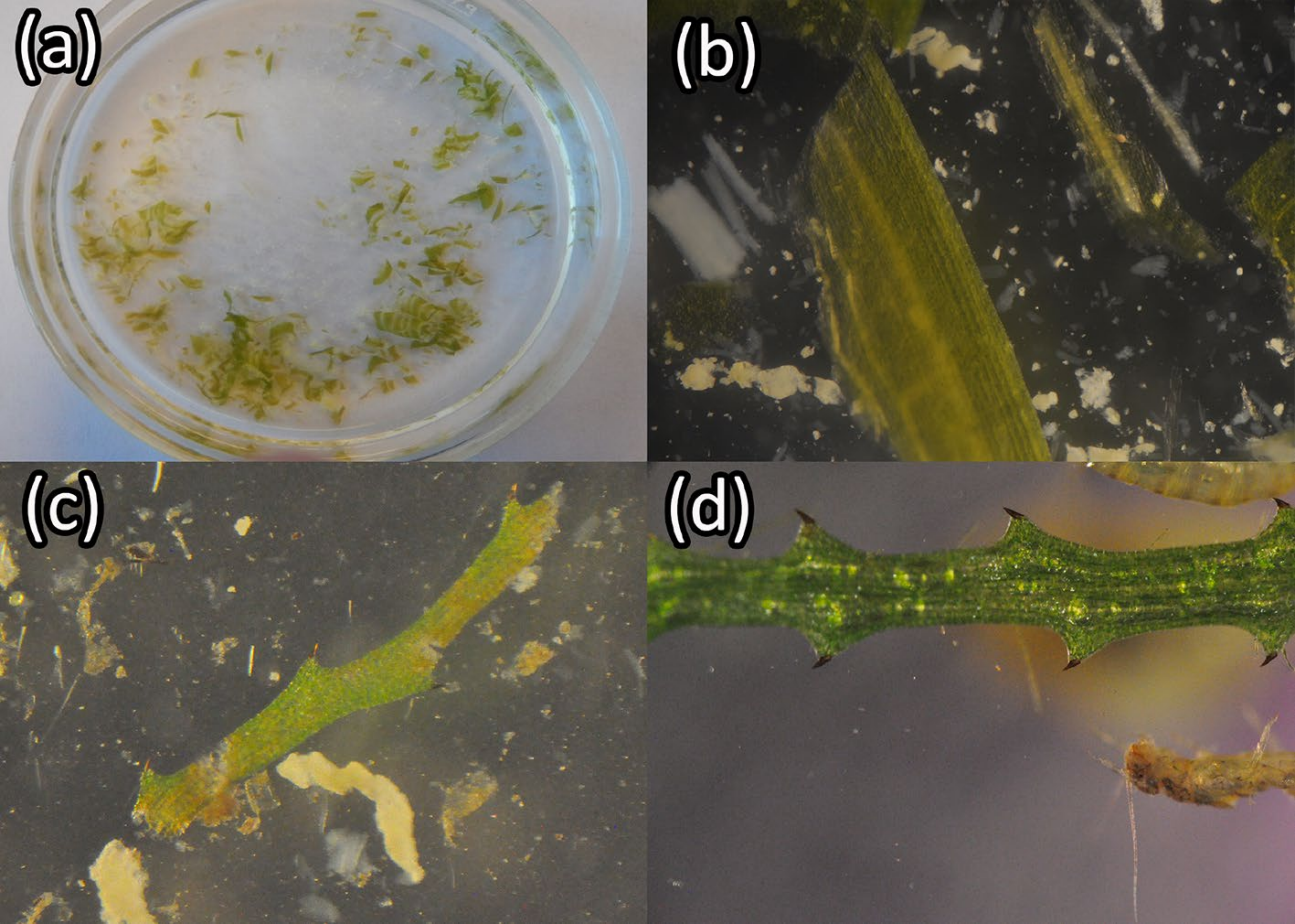
Plant matter within the stomachs of blue crabs: **a** the stomach of a *C. sapidus* after 72 h in a tank with 4 Vallisneria americana transplants; **b** a magnified imagine of *V. americana* pieces found in the stomach of a *C. sapidus*; **c** a piece of Najas minor found in the stomach of a *C. sapidus* collected within the Chickahominy River; and **d** a photo of a freshly collected piece of N. minor

The lengths of unprotected *V. americana* transplants (*β* = 54 ± 0.67, *P *< 0.001) and transplants in cages containing one *C. sapidus* (*β* = 25 ± 0.67, *P *< 0.001) were significantly different from the lengths of transplants in control cages without *C. sapidus* after 72 h (Table 1, Fig. 5). Dunnett’s comparisons indicated significant differences in final shoot lengths (*t*_d_ = − 2.9, *df* = 84, *P *= 0.009) between transplants from cages containing *C. sapidus* (least squares mean CI 4.4–7.8) and transplants outside any enclosure (open controls) (least squares mean CI: 6.8–10). Clipped transplants removed from cages containing *C. sapidus* appeared similar, however, to clipped transplants exposed to the entire herbivore community in the open water (Fig. 2b and Fig. 3b). Tessellated darters (*Etheostoma olmstedi*), mud crabs (likely *Rhithropanopeus* sp.), brackish water clams (*Rangia cuneata*), various amphipod species, and small juvenile *C. sapidus* (~ 1 cm CW) were observed in *C. sapidus* and control cages. No significant relationship was detected between the difference in total transplant leaf length within a given cage to the estimated volume of plant matter in a *C. sapidus* stomach after a cage trial (*P *= 0.1, Fig. S5). Plant matter was, however, present in 17 of the 18 dissected *C. sapidus* stomachs and was on average 46% of the estimated stomach volume of caged *C. sapidus* after 72 h (Fig. 6a, b).

**Table 1:** A summary table for a linear mixed effects model fit evaluating differences in the length of *V. americana* shoots remaining after 72 h in (caged control) and out (uncaged control) of cages and in cages with (crab) and without (caged control) crabs

Variables	Variable level	Estimate	SE	*df*	*p*
Crab treatment	Caged control	–	–	–	–
	Crab	25	0.67	83.9	< 0.001**
	Uncaged control	54	0.67	83.9	< 0.001**
Crab size	Small (≤ 8 cm)	–	–	–	–
	Large (> 8 cm)	1.2	1.3	4.9	0.4

*p < 0.05

**p < 0.001

### Non-native SAV consumption

**Fig. 7:**
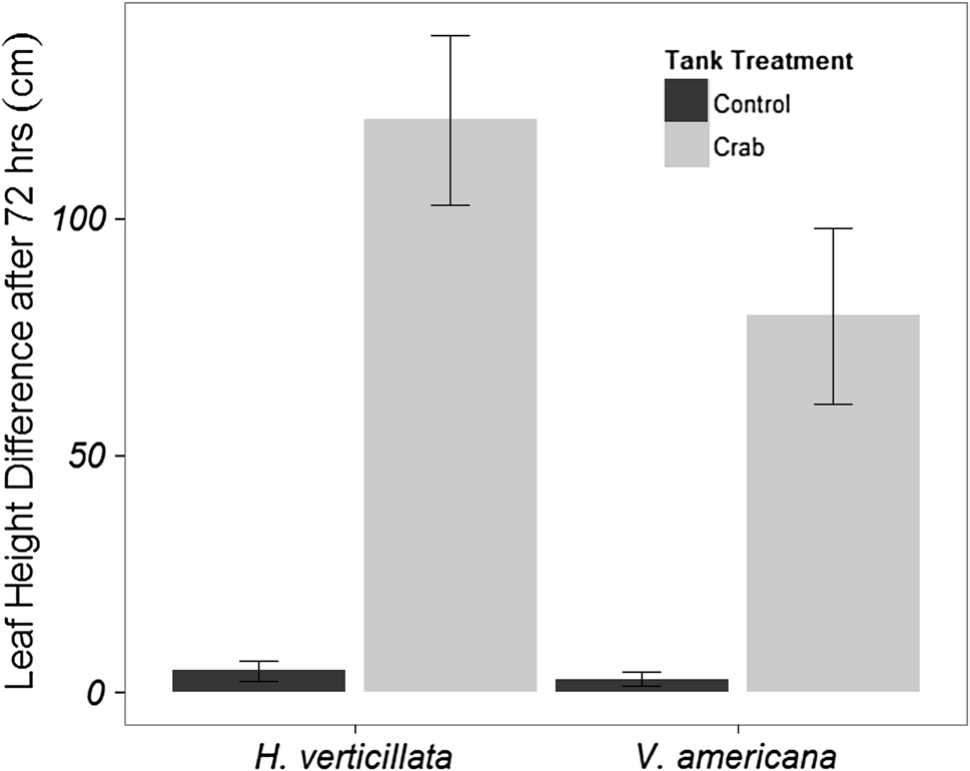
The mean difference in leaf lengths per tank (± SE) for *H. verticillata* (*n* = 16) or *V. americana* (*n* = 14) after 72 h with or without a *C. sapidus* in the tank

Transplants of *V. americana* and *H. verticillata* decreased significantly in shoot length after 72 h in tanks with *C. sapidus* relative to transplants in tanks without *C. sapidus* (*β* = 44.7 ± 1.44, *P *< 0.001, Fig. 7). No significant differences in total shoot length were detected between tanks planted with different transplant species (*p* = 0.6). Tearing or bite marks were more likely to be found on *V. americana* leaves in tanks with *C. sapidus* (odds ratio: 10.5, 95% CI 1.5–73, *p* < 0.001, Fig. 3c and S6) than in tanks without *C. sapidus*. Although no formal categorization of tear or bite marks was conducted for *H. verticillata* shoots, *H. verticillata* shoots were stripped of leaves in tanks containing *C. sapidus* on several occasions (Fig. 3d). The difference in total shoot length for a given tank was not significantly related to the estimated percentage of plant matter in a *C. sapidus* stomach (*β* = 0.2 ± 0.1, *P *= 0.05, Fig. S7 and S8) and clipped shoots were observed floating within tanks (Fig. S9).

### *Callinectes sapidus* diet survey

**Fig. 8:**
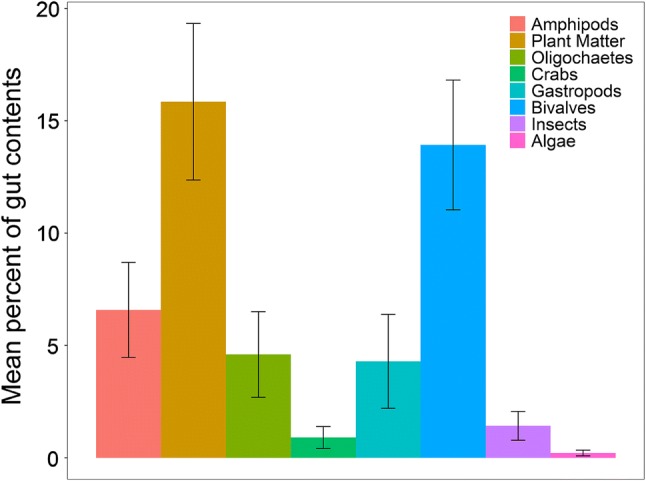
The mean percentage of identifiable food items (± SE) in the guts of *C. sapidus* collected from seine nets in the James and Chickahominy Rivers in 2017 (*n* = 52)

The majority of stomach volume (on average 44%) of dissected *C. sapidus* consisted of unidentifiable material. Plant matter was present in 32 of the 52 collected *C. sapidus* (61%) and was on average 16% of stomach contents (Figs. 6 and 8). Bivalves were the second most prevalent, identifiable food item, contributing on average 14% of stomach contents.

## Discussion

Our results provide an important example of how plant community structure and re-establishment, especially in an estuarine system, may be affected by herbivory of vulnerable, colonizing propagules that are important to the recruitment and dynamics of plant populations either through natural colonization or active restoration (Janzen 1970, 1971, Harper 1977). We have demonstrated using field observations and field and laboratory experiments that *C. sapidus* affects both native and non-native vegetation in the oligohaline waters of Chesapeake Bay by both clipping and consuming these plants. This behavior, which removes photosynthetic tissue from propagules, was found to occur consistently in both the James and Chickahominy Rivers. Grazing of this nature was previously demonstrated to prevent the initial recovery of one historic, native plant (Moore et al. 2010), but has not prevented the emergence and persistence of other non-native SAV species. The combined results of the current and previous studies suggest that herbivory, likely from a generalist, marine omnivore, *C. sapidus*, could act as the bottleneck to population recovery of a native species but not necessarily the non-native species in the area. This outcome may be related to the higher reproductive, growth, and dispersal mechanisms of the non-native vegetation which allows them to persist despite herbivory.

### *Callinectes sapidus* herbivory

This study is the first to document targeted consumption of submersed vegetation by an estuarine omnivore, *C. sapidus*, under experimental and natural settings. While variability in the prevalence of SAV in stomachs among the individuals observed here was large, these observations in combination with previous diet studies indicate that the contribution of plant matter to the diet of *C. sapidus* could be 4–29% (Laughlin 1982; Alexander 1986; Wolcott and O’Connor 1992; Seitz et al. 2011). Previous studies have demonstrated that *C. sapidus* may derive nutritional value from vegetation (McClintock et al. 1991). Because *C. sapidus* is ubiquitous and extremely common (it is one of the most valuable commercial fisheries in the Chesapeake Bay) in low-salinity estuarine regions throughout their range (Posey et al. 2005; Seitz et al. 2003), they could play a role in regulating population dynamics of SAV and other plant populations both here, and in many other areas where they co-exist (Alexander 1986). In addition, *C. sapidus* may be yet another of a large and diverse group of animals, from sea urchins and sea turtles to deer and sharks, that can derive some portion of their diet from submersed aquatic vegetation (Thayer et al. 1984; Eklӧf et al. 2008; Fourqurean et al. 2010; Ceacero et al. 2014; Leigh et al. 2018).

The clipping of single *V. americana* plants spaced at half-a-meter intervals from one another observed in this study suggests that *C. sapidus* feeds opportunistically on sparse shoots. Other known herbivores in the system, such as migratory waterfowl, muskrat (*Ondatra zibethicus*), or red bellied turtles (*Pseudemys rubriventris*), may seek larger stands of vegetation which will provide them a higher foraging efficiency than isolated shoots (Spongberg and Lodge 2005). Crayfish also have been shown to clip and consume *V. americana* in freshwater habitats (Lodge and Lorman 1987), but none were observed in this oligohaline system. Although additional herbivores are likely present in the James and Chickahominy Rivers, their abundance and influence were not detected in this or previous studies (Meier 2002).

Observations of clipped but unconsumed leaf material floating within experiments, as well as clipped and heavily damaged leaves, support an opportunistic herbivory hypothesis for *C. sapidus*, but also suggest that *C. sapidus* may “sample” SAV and then either partially or totally consume clipped plant material. In this study *C. sapidus* most commonly clipped leaves at their base and clipped every leaf from a shoot in most instances. Interestingly, some *C. sapidus* in experimental tanks may have torn or bitten sections of leaves (Fig. 3c, d) without clipping the entire shoot or leaf at the base. These observations, the variability in the abundance of plant matter among *C. sapidus* stomachs, and the difference in clipping between transplant leaves in cages with one *C. sapidus* and transplant leaves exposed to the entire herbivore community offer evidence that some *C. sapidus* may consume SAV more than others. The size of *C. sapidus* individuals and other unexplored variables, for example, alternative food availability, may explain the variability in *C. sapidus* vegetation consumption. Although epiphytes were initially removed from all vegetation used in experiments in this study, epiphyte growth could also lead to accidental grazing of SAV. The abundance of *M. leucophaeata* and other species both growing on vegetation and found within the stomachs of *C. sapidus* collected in the system (Table S3 and Fig. S10a and b) suggests that incidental damage and consumption of SAV may occur and could explain damage to vegetation without consumption of the vegetation (Video S2). Despite the potential for *C. sapidus* scavenging for epiphytes to damage SAV, the photographic and diet observations in this study clearly demonstrate that some *C. sapidus* consume vegetation.

Surprisingly, the non-natives *H. verticillata* and *N. minor* also appeared as an important component of the *C. sapidus* diet (16%), in addition to epifauna and infauna found in these meadows, e.g., mussels (*Mytilopsis leucophaeata*), gastropods (*Lymnea* spp.), and amphipods (*Corophium* sp.). Our diet data reveal the value of these non-native SAV communities to *C. sapidus* populations within the oligohaline portions of the lower Chesapeake Bay, and possibly elsewhere where they occur.

### Persistence of SAV with herbivory

Numerous studies in terrestrial and aquatic environments have shown that herbivores can alter the structure and composition of plant communities (Cyr and Pace 1993; Hanley 1998; Bakker et al. 2016). Our results in an aquatic environment demonstrate that *C. sapidus* consumes all studied SAV species, yet observations in the Chickahominy River found an abundance of *N. minor* and *H. verticillata* in the vicinity of experiments. Indeed, much of the shallow water areas of the Chickahominy River and many other low-salinity regions of the Chesapeake Bay maintain dense cover of these two species, and sometimes *V. americana,* despite the presence of *C. sapidus* (Orth et al. 2017). The reproductive potential and dispersal characteristics of each SAV species, the presence of water quality conditions suitable for rapid SAV growth and expansion, and the foraging behaviors of herbivores, such as *C. sapidus*, may help to explain the composition of SAV communities in the James and Chickahominy Rivers.

All three SAV species reproduce sexually, producing large numbers of seeds, and asexually, through rhizome or stolon extension (Langeland 1996, McFarland and Shafer 2008, Les et al. 2015). Propagule production and supply, however, differ among the three. For canopy-forming species, such as *N. minor* and *H. verticillata*, vegetative fragments clipped or ripped away from the parent plant are often shoots that can disperse and re-root to colonize new habitat (Rybicki et al. 2001). In many cases, the clipping or cutting of *H. verticillata* shoots has been found to only temporally reduce their abundance and regrowth occurs rapidly (Langeland 1996). However, for *V. americana*, a meadow-forming species whose leaves grow into the water column from a shoot in the sediment, clipped or torn vegetative fragments are often leaf material not capable of surviving and colonizing new habitats. Thus, herbivory, particularly from *C. sapidus*, can generate new propagules of *N. minor* and *H. verticillata*, but not so with *V. americana*. As a result, herbivory of very sparse SAV could further suppress propagule production of *V. americana* compared to these other SAV species.

The presence of large, dense stands of *V. americana* in the upper Chesapeake Bay (Orth et al. 2017) and other areas despite the presence of *C. sapidus* suggests that *V. americana* populations can overcome herbivore pressure. Future research should explore whether *C. sapidus* or other herbivores target SAV propagules in other systems (Fig. S12) and whether the proximity and diversity of SAV communities, additional propagule availability, or fluctuations in herbivore intensity allow establishing *V. americana* populations to overcome grazing pressure.

## Conclusions

Our results demonstrate that *C. sapidus* can remove photosynthetic tissue and consume SAV for small to moderate amounts as part of their diets in oligohaline environments. For some SAV species such as *V. americana*, herbivory, likely from *C. sapidus*, could prevent population re-establishment in areas with low SAV propagule availability. Although we have shown that *C. sapidus* also consume other SAV species, including *N. minor* and *H. verticillata*, the capacity of these SAV species to reproduce and spread rapidly using both seeds and vegetative propagules may allow them to colonize available habitats and overcome this grazing pressure limitation. Reductions to herbivore populations, increased propagule production and dispersal through restoration efforts (Orth et al. 2012), and direct exclusion of herbivores from restored, founder beds (Moore et al. 2010) may all be necessary for some species populations to reach the size and abundance necessary to overcome herbivory bottlenecks and become self-sustaining.

## Electronic supplementary material

Below is the link to the electronic supplementary material.

Supplementary material 1 (DOCX 3195 kb)
