# Supplementary material for: Herbivory regulates the establishment of a native species of submerged aquatic vegetation (SAV) in a tidal estuary of the USA
Source: Oecologia. 2019 Jun 22;190(3):639–50. doi: 10.1007/s00442-019-04439-4 (PMC6647907; doi:10.1007/s00442-019-04439-4)
Supplement: Supplementary file 1 — Supplementary material 1 (DOCX 3195 kb) [file 442_2019_4439_MOESM1_ESM.docx]

**Electronic Supplementary Material for the manuscript:**

**Herbivory regulates the establishment of a native species of submerged aquatic vegetation (SAV) in a tidal estuary of the USA**

A.J. Johnson^*^, R.J. Orth, K.A. Moore

^1^Department of Biological Sciences, Virginia Institute of Marine Science, College of William & Mary, Gloucester Point, VA 23062, USA

*Corresponding author at: Virginia Institute of Marine Science, PO Box 1346, Gloucester Point, VA 23062-1346, USA. E-mail address: [ajjohnson@vims.edu](mailto:ajjohnson@vims.edu)

**Video S1**

[**https://drive.google.com/file/d/1KVOhs0kMTQKb_zwIO6Hpit89VyXWQSqc/view?usp=sharing**](https://drive.google.com/file/d/1KVOhs0kMTQKb_zwIO6Hpit89VyXWQSqc/view?usp=sharing)

**Video S1. Video of blue crab consumption of *V. americana* (bottom right of screen)**

**Video S2:**

<https://drive.google.com/file/d/13HF_nrLXk-nqvyR78dHIv5EukE8BwSHw/view?usp=sharing>

**Video S2.** An uploaded video of a blue crab consuming epiphytes off the leaves of *Zostera marina* in the lower Chesapeake Bay, VA. This consumption behavior could potentially damage or tear leaves of SAV and is worthy of further investigation.

**Table S1**

**Table S1.** The species identified within the field of view from 78 hrs of time series photography conducted in the James and Chickahominy Rivers, Virginia, from August – September 2017. All animals moving into the field of view which could not be identified were categorized a “Unidentifiable.”

| Species | Scientific Name | Number of Observations |
| --- | --- | --- |
| Juvenile Sunfish | *Lepomis* sp*.* | 42 |
| Spot | *Leiostomus xanthurus* | 22 |
| Blue Crab | *Callinectes sapidus* | 17 |
| Tessellated Darter | *Etheostoma olmstedi* | 16 |
| Bay Anchovy | *Anchoa mitchilli* | 1 |
| Blue Catfish | *Ictalurus furcatus* | 1 |
| Striped Bass | *Morone saxatilis* | 1 |
| American Eel | *Anguilla rostrata* | 1 |
| Unidentifiable | - | 106 |

**Table S2**

**Table S2.** A generalized linear model of grazing intensity of 20 transplants after 24 hours and 1 week along transects at two locations over three separate trials in late summer 2016. Asterisks indicate the statistical significance of coefficients at p < 0.05 (*) or p < 0.001 (**).

| **Variable** | **Coefficient** | **Std. Error** | **t-value** | **P - value** |
| --- | --- | --- | --- | --- |
| Duration: 1 Week | 9.3 | 1.7 | -3.9 | < 0.001** |
| Location: Chickahominy | 2.6 | 1.8 | 4.0 | 0.11 |
| Trial: Trial 2 | 0.37 | 1.5 | 1.6 | 0.01* |
| Trial: Trial 3 | 0.20 | 1.5 | -2.6 | < 0.001** |
| Duration*Location | 2.6 | 2.0 | -3.8 | 0.18 |
| Intercept | 0.14 | 1.6 | 1.3 | < 0.001** |

**Table S3:**

**Table S3.** Fauna identified within *Najas minor* meadows adjacent to the experimental area.

| Habitat | Species | | Number of Observations |
| --- | --- | --- | --- |
| Epifauna | *Lymnea* spp. | 116 | |
|  | *Mytilopsis leucophaeata* | 115 | |
|  | *Unidentified caddisfly** | 31* | |
|  | *Corophium* sp. | 18 | |
|  | Mayfly Nymphs (*Ephemeroptera)* | 10 | |
|  | *Gammarus spp.* | 3 | |
|  | *Lembos smithii* | 2 | |
|  | *Palamaentes pugio* | 2 | |
|  | *Syngnathus fuscus* | 1 | |
|  | Unidentifiable Amphipods | 6 | |
|  | Unknown tubeworm | 1 | |
|  | *Chironomidae* larvae | > 100 | |
| Infauna | *Tubifex* sp. | 5 | |

* Unidentified caddisfly was observed at far higher frequency along the stems of *Najas minor* within the system.

**Figure S1:**

**
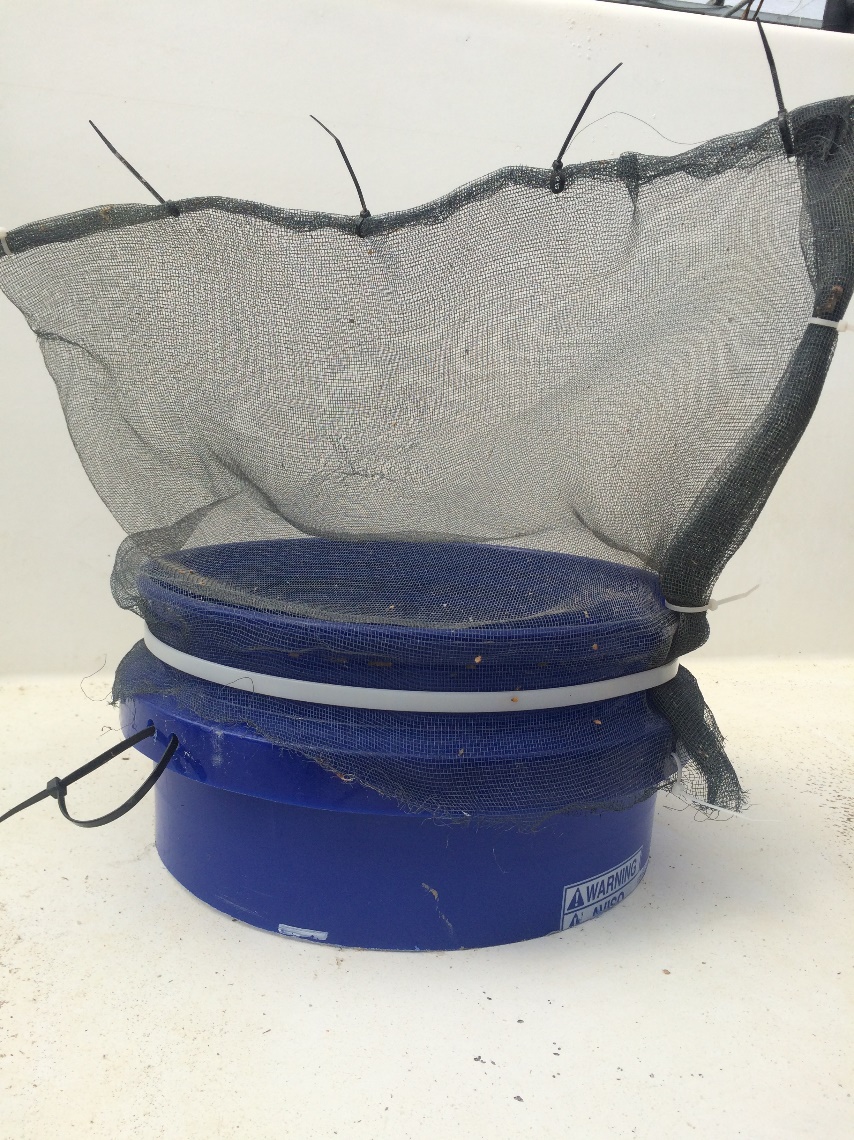
**

**Figure S1.** Aluminum wire cages used to exclude or contain blue crabs during *in situ* caging studies. The plastic bottom of each cage (in blue) was inserted into the sediment at least 7 cm. One 50 cm L, 5 cm W PVC stake and one 50 cm rebar stake were placed within plastic ties on either side of the bucket to anchor it in place.

**Figure S2:**


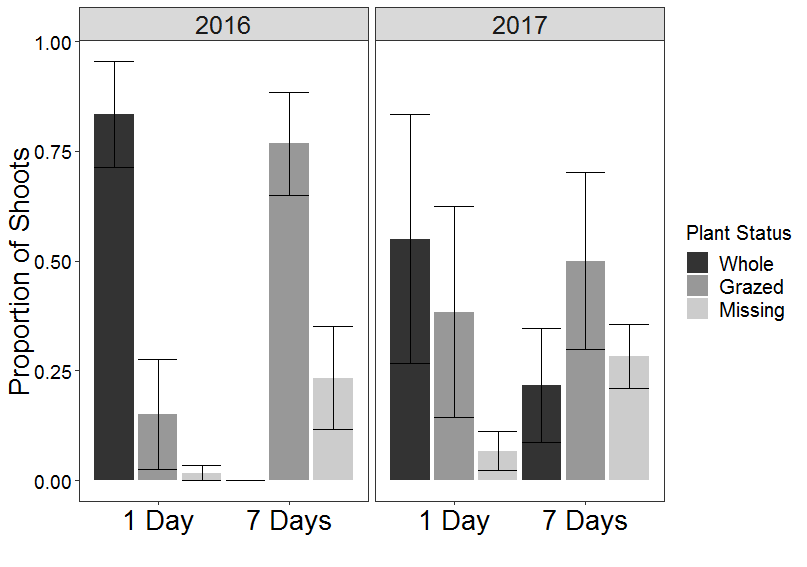


**Figure S2.** The proportion of shoots (± SE, n = 20) consumed within 24 hours and after one week along transects (n = 3) at the mouth of the Chickahominy River in 2016 and 2017.

**Figure S3:**


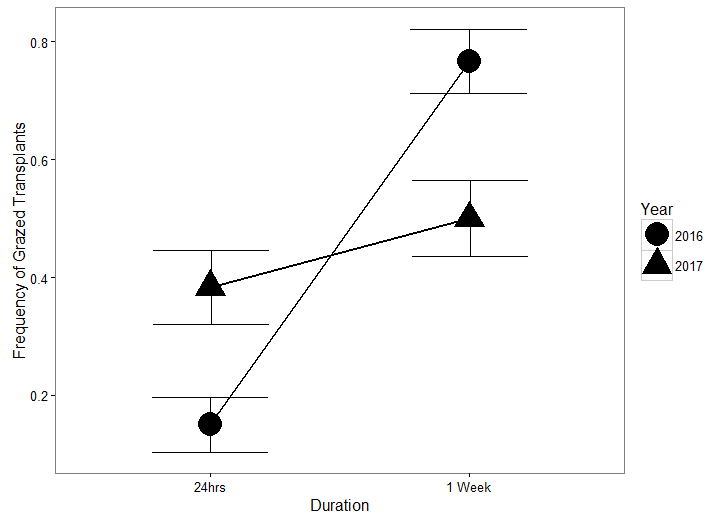


**Figure S3.** The mean frequency (± SE) of grazed transplants (n = 3 transects) observed in late summer of 2016 and 2017.

**Figure S4:**


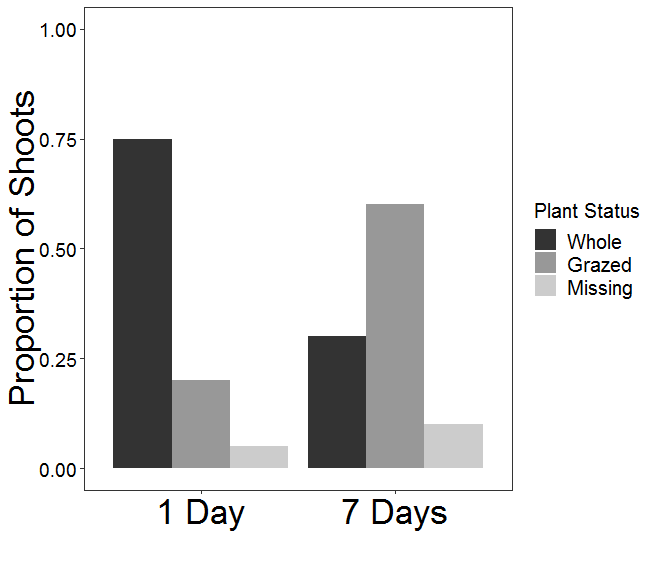


**Figure S4.** The proportion of Vallisneria americana transplants (n = 20) clipped within a Najas minor meadow adjacent to transect lines in summer 2016.

**Figure S5**


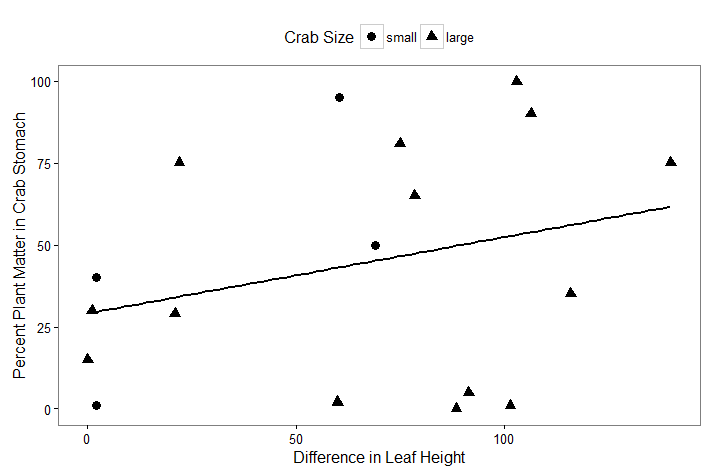


**Figure S5.** A scatterplot of the difference in transplant leaf height and the percent of plant matter in crab stomachs for each in situ caging trial for which the crab could be recovered with a non-significant trend line (p = 0.1). Small crabs (CW < 8 cm) are displayed with circles and large crabs (> 8 cm) are displayed with triangles.

**Figure S6**


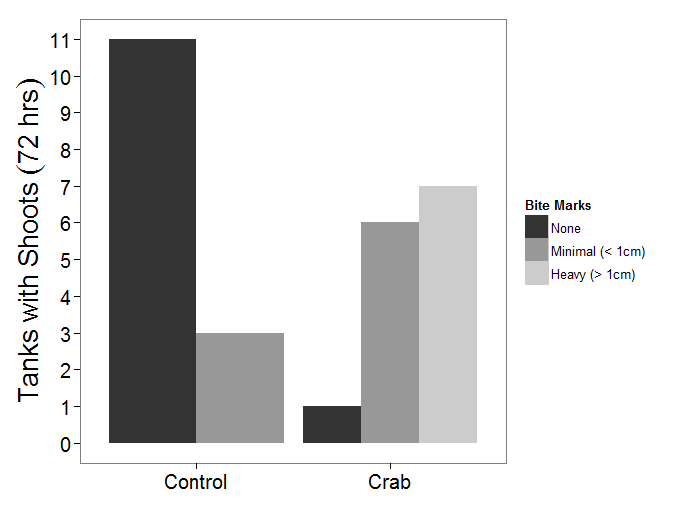
**Figure S6.** The frequency of bite mark categories observed on *V. americana* shoots from tanks with and without blue crabs.

**Figure S7:**

**
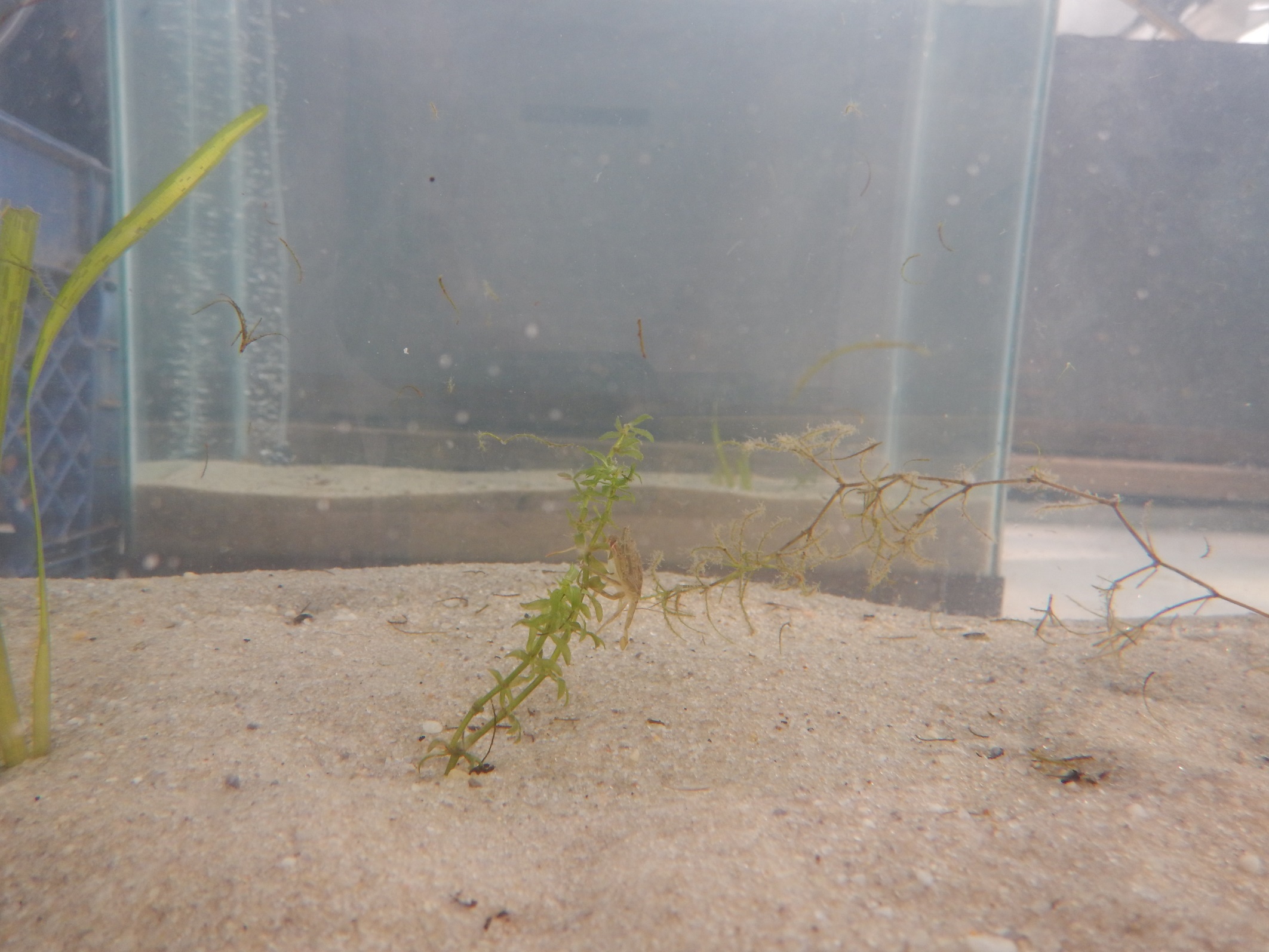
**

**Figure S7.** A juvenile blue crab clipping the leaves of a Hydrilla verticillata propagule in a preliminary feeding experiment. Blue crabs in these preliminary trials also consumed Najas minor transplants (pictured in the background), a finding stomach contents of wild-caught blue crabs corroborates.

**Figure S8**


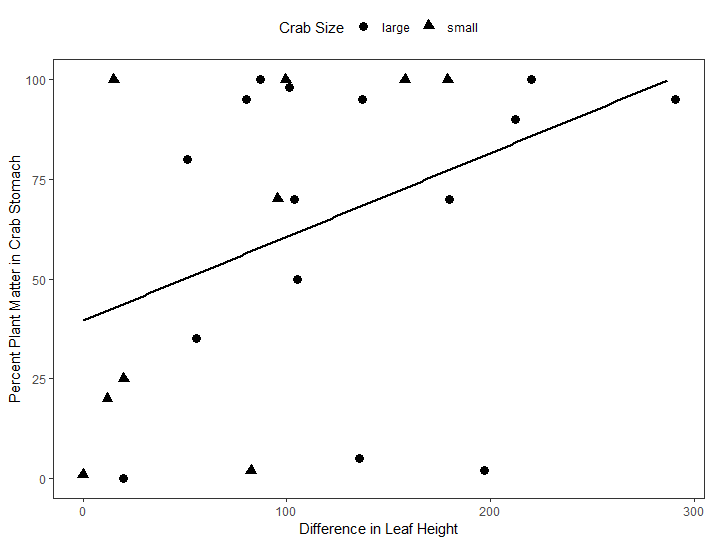


**Figure S8.** A scatterplot of the difference in total leaf height and the percent of plant matter in crab stomachs (n = 24) for each tank during non-native grazing experiments with a non-significant trend line (p = 0.05). Small crabs (CW < 8 cm) are displayed with circles and large crabs (> 8 cm) are displayed with triangles.

**Figure S9**


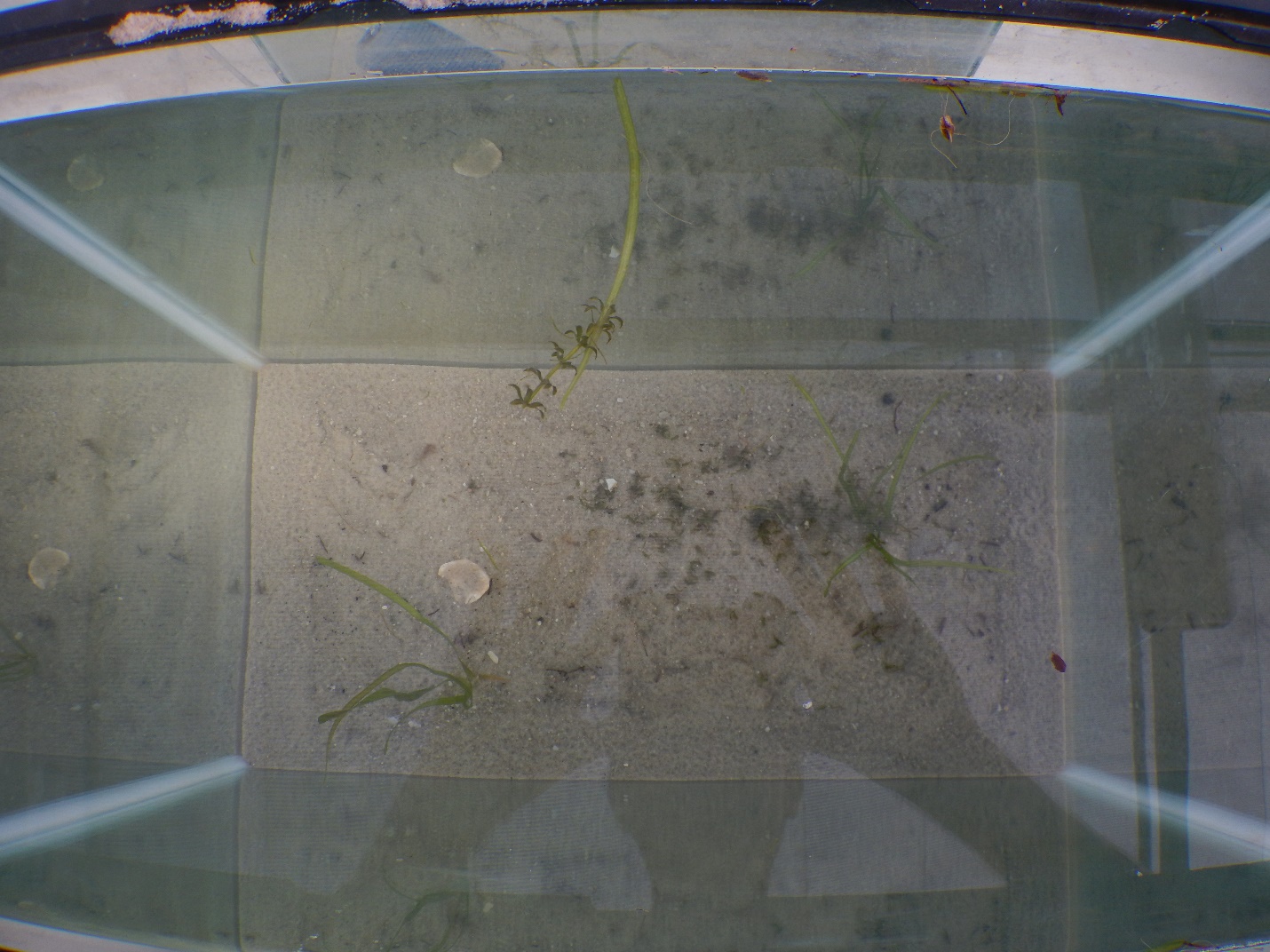


**Figure S9.** Floating fragments of Vallisneria americana (circled in blue) and Hydrilla verticillata (circled in red) clipped by Callinectes sapidus during a preliminary feeding trial. Dark matter on the sand at the bottom of the tank were clipped leaves of H. verticillata.

**Figure S10:**


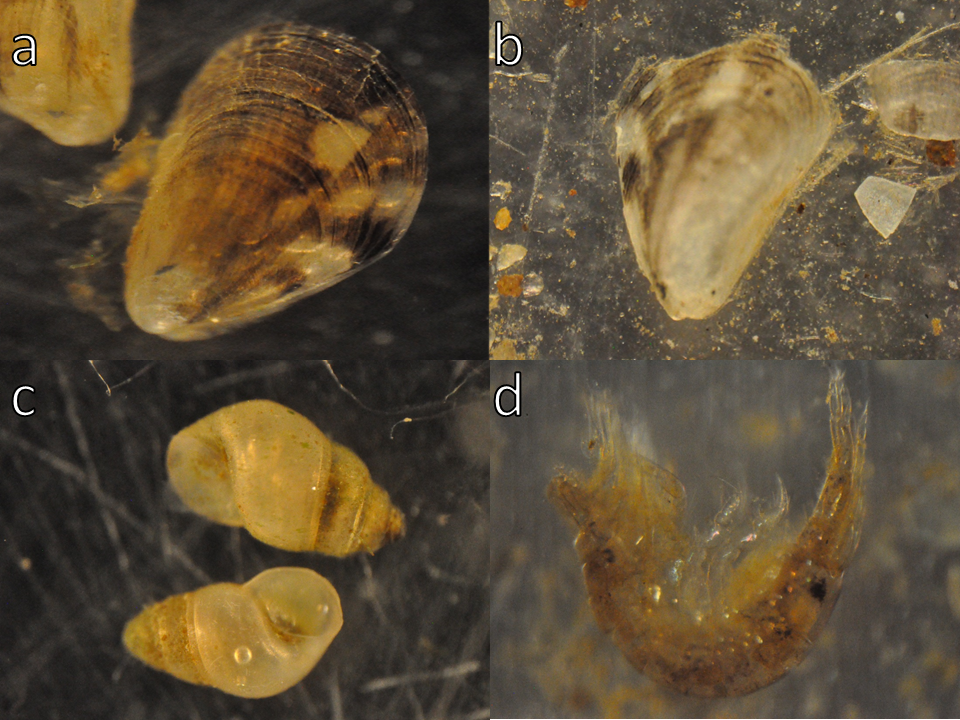


**Figure S10.** Prey in the stomachs of blue crabs collected within a meadow of non-native vegetation : (a) A shell of Mytilopsis leucophaeata from an epifaunal grab bag sample; (b) a magnified imagine of a M. leucophaeata shell found in the stomach of a blue crab collected from the Chickahominy River; (c) the shells of Lymnea spp. gastropods collected from Chickahominy River and identified in blue crab stomachs; and (d) a Corophium spp. amphipod found in the stomach of a blue crab and within epifaunal samples from non-native vegetation in the Chickahominy River.

**Figure S11:**


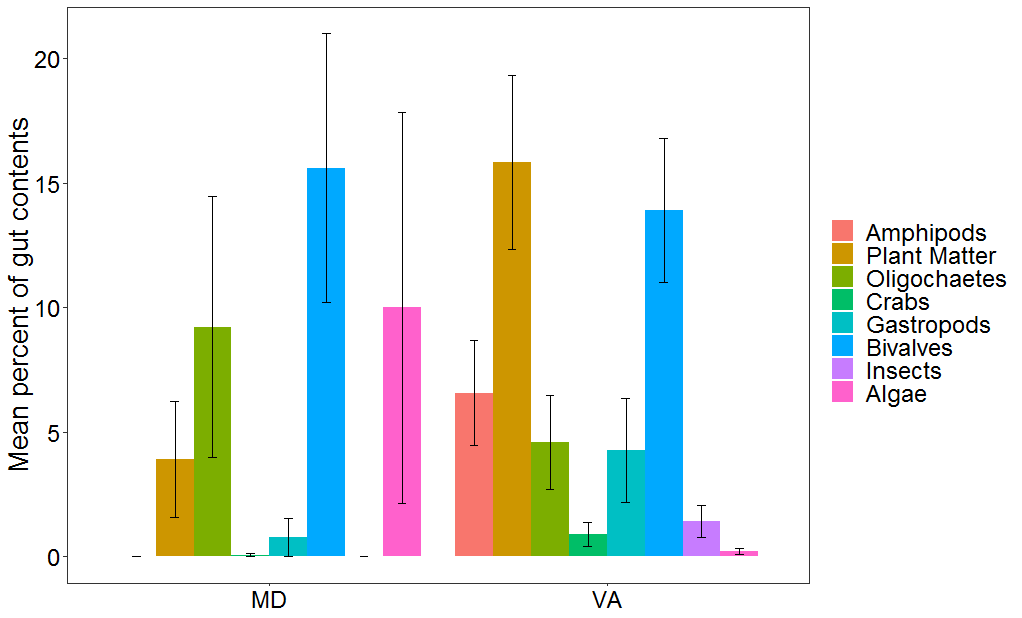


**Figure S11.** The mean percentage of identifiable food items in the guts of blue crabs collected in the lower Chesapeake Bay (VA, n = 52) and upper Chesapeake Bay (MD, n = 13). To evaluate whether the diets of blue crabs differed significantly in tidal freshwater areas with established V. americana populations to areas where V. americana has not recovered, the stomach contents of blue crabs captured at locations with V. americana in the upper Chesapeake Bay (Maryland) were compared to the stomach contents of blue crabs in the lower Chesapeake Bay (Virginia) where V. americana populations have not recovered. Maryland crabs were caught from two locations in the Elk River and one within the Susquehanna Flats; both locations of extensive SAV meadows with abundant V. americana populations. Plant matter was found in blue crab stomachs from both locations, but a considerable range in plant and overall diet may exist between locations.

**Figure S12:**


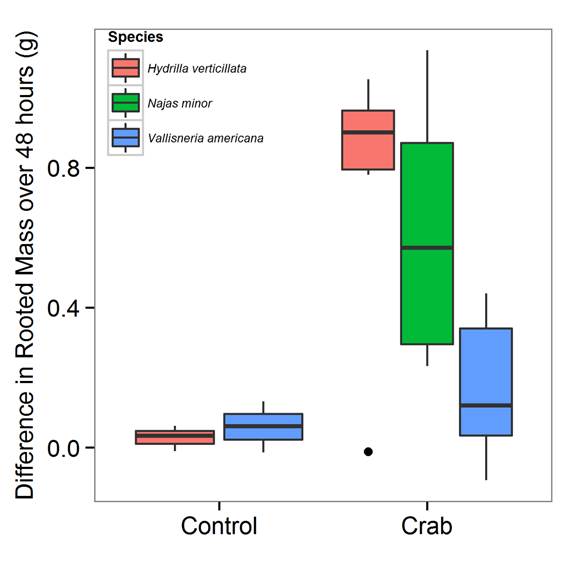


**Figure S12.** The difference in biomass of SAV species left with a single blue crab for 72 hours in a tank environment. Plants were dried in a salad spinner according to a fixed protocol before weighing. This preliminary study was not conducted in a temperature controlled water bath, so was not included in the main study as temperature fluctuations could impact blue crab behavior. In addition, blue crab clipping, consumption, and damage to plant matter made recovery and drying of all pieces of vegetation after blue crab exposure difficult. Last, we did not have sufficient N. minor to plant in control tanks. As a result, any statistical comparison for this species would be impossible. We include this figure simply to demonstrate blue crabs damaged, clipped, or consumed all three species of SAV featured in the study. Boxes and whiskers indicate data quartiles and dark bars across boxes indicate data medians.
